# Comparison of substernal and posterior mediastinal route of reconstruction after minimally invasive esophagectomy for esophageal cancer

**DOI:** 10.1007/s00423-023-03215-6

**Published:** 2024-01-06

**Authors:** Tran Quang Dat, Dang Quang Thong, Doan Thuy Nguyen, Nguyen Viet Hai, Nguyen Hoang Bac, Vo Duy Long

**Affiliations:** 1 Gastro-intestinal Surgery Department, University Medical Center, University of Medicine and Pharmacy at Ho Chi Minh City, 215 Hong Bang, Ward 11, District 5, Ho Chi Minh City, Vietnam; 2 Department of General Surgery, Faculty of Medicine, University of Medicine and Pharmacy at Ho Chi Minh City, Ho Chi Minh City, Vietnam

**Keywords:** Esophageal cancer, McKeown minimally invasive esophagectomy, Substernal route, Posterior mediastinal route

## Abstract

**Background:**

Substernal (ST) and posterior mediastinal (PM) routes are the two most common for reconstruction after esophagectomy with cervical anastomosis. Recent evidence showed similar outcomes between the routes; thus, the superior choice remained controversial. This study aimed to compare the short-term outcomes of the ST to the PM route for reconstruction after esophagectomy for esophageal cancer (EC).

**Method:**

This retrospective cohort study included 132 patients who underwent McKeown minimally invasive esophagectomy (MIE) with gastric conduit for EC between March 2015 and December 2022. Among these, 89 and 43 patients received the ST route and PM route for reconstruction, respectively. Short-term outcomes including operative characteristics, postoperative morbidity, and mortality were evaluated.

**Result:**

There was no conversion from ST to PM route. The ST group had longer operating time (375 min vs. 341 min). Oral feeding initiation, postoperative hospital stays, and overall complication rates were comparable in the two groups. The rate and severity of anastomotic leakage were similar between the groups. The ST group had a significantly lower incidence of postoperative ICU admission and pneumonia compared to the PM group (5.6% vs. 16.3% and 19.1% vs. 37.2%, respectively). Azygos vein bleeding, obstruction at feeding jejunostomy site, and conduit–trachea fistula were severe complications that only occurred in PM route.

**Conclusion:**

ST route was superior to PM route in term of postoperative ICU admission and pneumonia. This route may prevent severe complications that only occur in PM route. ST route can be favorable option for reconstruction after McKeown MIE for EC.

## Introduction

Esophageal cancer (EC), the seventh most common cancer worldwide, is associated with a high incidence of mortality [1]. Despite significant progress in multimodality treatment, the prognosis remains unfavorable, with a 5-year overall survival rate of 4.1–42% [2]. Esophagectomy with extended lymph node dissection is the primary treatment for thoracic EC and is associated with an overall complication rate of up to 60%, even when conducted in high-volume centers [3].

Esophagectomy by McKeown is the most common type of esophagectomy for EC due to its benefits in curability and long-term outcomes [4]. Recently, minimally invasive esophagectomy (MIE) has become standard treatment for this cancer owing to numerous advantages such as reduced postoperative complications, enhanced recovery, and improved survival rate [5].

The stomach is the most common organ used for alimentary reconstruction via the posterior mediastinal (PM) or substernal (ST) route. Both routes have advantages and disadvantages based on the results of previous studies [6–8]. Anastomosis-related and pulmonary complications appear directly associated with the reconstruction routes.

The advantage of PM route is the unnecessariness to create an artificial compartment. In the other hand, gastric conduit placed in the ST route would not be affected by mediastinal recurrence or radiation therapy [8, 9]. Moreover, reconstruction through the ST route might help avoid some severe complications such as airway-gastric conduit fistula [10], hiatal and para-conduit hernia [11, 12], and it might reduce incidences of gastroesophageal reflux [13], and pulmonary complications, particularly pneumonia compared with the PM route [14]. Meanwhile some prior studies showed a lower anastomotic leakage rate using PM route [15–18], the others revealed similar rates of anastomotic leakage between the two groups [11, 19]. Two meta-analyses conducted on this issue showed no difference in postoperative outcomes for the two routes of reconstruction [8, 20]. Consequently, the superior choice between PM and ST route after esophagectomy for EC remains controversial. We conducted this study to compare the ST and PM routes after MIE in terms of intra- and postoperative complications and short-term outcomes for treating thoracic EC.

## Patients and method

### Patients

This was a retrospective cohort study reviewing patients who underwent McKeown MIE for EC between March 2015 and December 2022 at the Department of Gastrointestinal Surgery, University Medical Center, the tertiary hospital in Ho Chi Minh City, Vietnam. The inclusion criteria were as follows: (1) histologically confirmed squamous cell carcinoma or adenocarcinoma of the esophagus, (2) patients underwent thoracoscopic McKeown esophagectomy with cervical anastomosis, and (3) gastric conduits were used for reconstruction through PM or ST routes. We excluded patients with (1) an American Society of Anaesthesiology (ASA) score of 4, (2) a history of previous thoracic surgery, and (3) complications such as bleeding or perforation required for emergency esophagectomy. The study was approved by the Institutional Review Board of the hospital.

We started performing McKeown minimally invasive esophagectomy using the posterior mediastinal route since 2008 and the substernal route since 2015. The determination of the reconstruction route relied on the surgeons’ preferences and oncological factors. We preferred using the substernal route for patients with advanced tumors, who was candidates for adjuvant radiochemotherapy and was at risk of local recurrence.

### Surgical approach

MIE was performed in accordance with esophageal cancer practice guidelines by the Japan Esophageal Society [21]. The patient was placed in the prone or semi-prone position under left one—lung ventilation using double—lumen endotracheal intubation [21, 22]. Four or five trocars were placed. Firstly, the azygos vein was clipped by Hem-o-lok and then transected. The esophagus was then mobilized from the thoracic inlet to the diaphragmatic reflection and transected by a linear stapler with a proximal margin of at least 5 cm. Next, lymph node dissection was performed with extension depending on the tumor location, depth of invasion, and histological type. Finally, a thoracic drainage tube was inserted.

In the abdominal phase, the stomach mobilization and abdominal lymphadenectomy were performed via laparoscopy or laparotomy. The right gastroepiploic and the first branches of the right gastric vessels were preserved for the blood supply of gastric conduit. The abdominal lymph node dissection included stations 1, 2, 3a, 7, 8a, 9, 11p, 11d, and para-esophageal LNs. Subsequently, a gastric conduit was created along the lesser curvature using linear staplers.

In the cervical phase, a left-cervical incision was made, and part of the sternothyroid muscle was incised to access the thoracic inlet without dissection of the clavicle. For the PM route, the gastric conduit was pulled through the posterior mediastinal route by attaching it to a tube. For the ST route, the proximal thoracic inlet (between the cervical and posterior mediastinal cavities) and the distal inlet (esophageal hiatus) were routinely closed. A substernal space was created from the epigastric incision to the neck using blunt dissection. This space was widened to accommodate the gastric conduit properly.

Finally, a side-to-side esophagogastric anastomosis was carried out using a linear stapler. Cervical drainage was not placed routinely. A feeding tube (gastrostomy in the ST route and jejunostomy in the PM route) was inserted for early postoperative enteral nutrition.

### Postoperative treatment

Antibiotics were administered for 5–7 days, and thoracic drain was removed within 2–3 days after surgery. The use of nasogastric tube was not standard practice. The patients were administered enteral feeding on the postoperative day 2, oral fluid diet on the postoperative day 4, and solid diet on postoperative day 6.

In my hospital, after surgery, patient spent 12–24 h in the Post Anesthesia Care Unit (PACU) before being transferred to the gastrointestinal surgery department. The patient was admitted to the ICU department due to one or more organ dysfunctions requiring specific management or pneumonia necessitating oxygen supplementation and specific monitoring.

### Outcomes

Perioperative data were collected including patient’s characteristics, tumor location, histologic type, tumor stage, status of lymph node metastasis, preoperative chemoradiation therapy, operative time, and intraoperative complications. Short-term outcomes included postoperative complications, time to oral intake, and postoperative length of stay. Postoperative complications were evaluated during hospital stay or 30 days after the surgery, including anastomotic leakage, ICU admission, pneumonia, pleural effusion required thoracic drainage, chylothorax, wound infection, cardiovascular complications, recurrent nerve injury, reoperation, and intrahospital mortality. Severity of complication was graded using Clavien-Dindo classification system [23]. The anastomotic leakage was diagnosed based on clinical or radiological signs.

The severity of cervical anastomotic leakage was graded based on the classification of Esophagectomy Complications Consensus Group (ECCG)[24]. We also collected data on the postoperative date (POD) of leakage. Pneumonia was diagnosed based on the following criteria: (1) presence of an infiltrative shadow on the chest X-ray or computed tomography, along with one or two of the parameters as follows, (2) presence of respiratory symptoms such as coughing and sputum…, and (3) an increased white blood cell count or a fever of > 38 °C. Postoperative pleural effusion which required drainage was recorded as a complication. Wound infection was diagnosed when purulent exudate was found in the wound. Postoperative mortality referred to any death occurring within 30 days after surgery or any death related to complications, irrespective of the timing of occurrence.

### Statistical analysis

Summary statistics were mean ± standard deviation or median (interquartile range) for quantitative variables and frequency and percentage for qualitative variables. Differences between the two groups were identified using the Mann-Whitney *U* test and Fisher’s exact test for quantitative and qualitative variables, respectively. All analyses were done with the statistical software Stata, version 17.

To investigate the risk factors of postoperative pneumonia, univariable analysis was performed using two sample *t*-test for normally distributed numeric variables, the chi-squared test or Fisher’s exact test for categorical variables, and the Wilcoxon rank sum test for non-normally distributed numeric variables. Multivariable analysis was conducted using logistic regression models with a stepwise backward procedure to identify independent risk factors of pneumonia.

## Results

### Patient’s characteristics

Between March 2015 and December 2022, a total of 132 patients who underwent McKeown MIE were included in this study. Among them, 89 patients underwent the ST route, and 43 patients underwent the PM route. The patient’s characteristics were summarized in Table 1. Baseline patient characteristics including gender, BMI, comorbidity, tumor location, albumin concentration, histologic type, preoperative chemoradiation, tumor stage, and lymph node metastasis status were balanced between the two groups. However, the mean age of patients in the ST group was significantly higher than in the PM group (60.9 years vs. 57.0 years, *p* = 0.006, respectively). The predominant comorbidities were hypertension (27.0% vs. 20.9%), followed by diabetes (12.4% vs. 4.7%) and chronic lung diseases (11.2% vs. 7.0%). The majority of tumor locations were the middle- and lower-third of the esophagus (80.8% vs. 88.3%). Most of the patients were diagnosed at locally advanced stages with T3-4 (76.4% vs. 81.4%) and lymph node metastasis (92.1% vs. 67.4%).

**Table 1:** Preoperative clinical characteristics

	Substernal (*N* = 89)	Posterior mediastinal (*N* = 43)	*p*-value
Age (years)	60.9 ± 7.8	57.0 ± 7.2	0.006
Sex male	84 (94.4)	40 (93.2)	0.759
BMI (kg/m^2^)	20.1 ± 3.2	20.4 ± 2.4	0.630
Hypertension	24 (27.0)	9 (20.9)	0.453
Diabetes	11 (12.4)	2 (4.7)	0.164
Cardiovascular disease	5 (5.6)	3 (7.0)	0.759
Chronic kidney disease	1 (1.1)	0 (0)	0.485
Chronic lung disease	10 (11.2)	3 (7.0)	0.442
Albumin (g/L)	39.2 ± 3.7	39.8 ± 3.2	0.433
Histology of disease			0.968
Squamous cell carcinoma	85 (95.5)	41 (95.3)	
Adenocarcinoma	4 (4.5)	2 (4.7)	
Neoadjuvant therapy	12 (24.7)	4 (9.3)	
Tumor location			
Cervical	1 (1.2)	2 (4.7)	0.085
Upper third	3 (3.4)	2 (2.7)	
Middle third	41 (46.0)	27 (62.8)	
Lower third	31 (34.8)	11 (25.5)	
EGJ	13 (14.6)	1 (2.3)	
Abdominal approach			0.128
Laparoscopy	85 (95.5)	38 (88.4)	
Laparotomy	4 (4.5)	5 (11.6)	
Pathological tumor stage			0.378
T1	5 (5.6)	0 (0)	
T2	16 (18.0)	8 (18.6)	
T3	44 (49.4)	20 (46.5)	
T4	24 (27.0)	15 (34.9)	
Pathological N stage			0.068
N0	7 (7.9)	14 (32.6)	
N +	82 (92.1)	29 (67.4)	

Summary statistics are mean ± sd and *n* (%)

*BMI* body mass index, *EGJ* esophago-gastric junction

### Operative characteristics

There was no conversion from ST route to PM route. The mean operating time was significantly shorter in the PM group than in the ST group (375 min vs. 341 min, *p* = 0.006, respectively). However, the rate of ICU admission was significantly lower in the ST group (5.6% vs. 16.3%, *p* = 0.046). Additionally, there were no statistical differences in terms of date of oral feeding and postoperative hospital length of stay (8.0 days vs. 7.9 days and 12.0 days vs. 13.7 days, respectively) (Table 2).

**Table 2:** Operative characteristics

	Substernal (*N* = 89)	Posterior mediastinal (*N* = 43)	*p*-value
Operation time	375.5 ± 66.8	341.1 ± 64.0	0.006
ICU admission	5 (5.6)	7 (16.3)	0.046
Postoperative length of stay	12.0 ± 4.6	13.7 ± 7.7	0.107
Date of oral feeding	8.0 ± 4.3	7.9 ± 1.8	0.938

Summary statistics are mean ± sd, *n* (%)

*ICU* intensive care unit

### Intra- and postoperative complications

Regarding intraoperative complications, one patient in the PM group experienced severe bleeding from the azygos vein that required conversion to thoracotomy. In this particular case, the Hem-o-Lok clip, which was used to ligate the azygos vein, was slipped out while attempting to pull up the gastric conduit towards the cervical cavity through the PM route.

The postoperative complications of the two groups are listed in the Table 3. The overall postoperative complications were similar between the two groups (44.9% vs. 34.9%). Most complications were classified as grade I or II according to Clavien-Dindo classification and did not differ between the groups. The major postoperative complications were also comparable (4/89 patients (4.5%) vs. 2/43 patients (4.7%)). Moreover, there were no significant differences in terms of the rate of anastomotic leakage (29.6% vs. 32.6%), severity of leakage (grade 1: 84.6% vs. 64.3%; grade 2: 7.7% vs. 28.6%, grade 3: 7.7% vs. 7.1%), and median date of leakage (POD 9 vs. POD 8) between the two groups. However, the ST group had a significant lower percentage of postoperative pneumonia compared to the PM group (19.1% vs. 37.2%, *p* = 0.024). The reoperation rate was similar in the two groups, 2.3% (2 patients) in the ST group versus 4.7% (2 patients) in the PM group. Each group had one case of reoperation due to anastomotic leakage. The reasons of reoperation of other patients were major chylothorax due to thoracic duct injury in the ST group, and acute intestinal obstruction at the site of feeding jejunostomy in the PM group. During the hospital stay, there were two deaths in the ST group due to acute myocardial infarction in one patient and septic shock because of anastomotic leakage in the other patient. One patient in the PM group died of septic shock due to a conduit–trachea fistula.

**Table 3:** Intra- and postoperative complications

	Substernal (*N* = 89)	Posterior mediastinal (*N* = 43)	*p*-value
Any complications	40 (44.9)	15 (34.9)	0.546
Clavien-Dindo classification*			0.517
I–II	36 (90)	13 (86.7)	
IIIa	0 (0)	0 (0)	
IIIb	1(2.5)	1 (6.7)	
IV	1(2.5)	0 (0)	
V	2 (5)	1 (6.7)	
Leakage	26 (29.6)	14 (32.6)	0.725
Date of leakage, median (IQR)	9 (5–17)	8 (7–9)	0.476
Serverity of leakage			0.209
1	22 (84.6)	9 (64.3)	
2	2 (7.7)	4 (28.6)	
3	2 (7.7)	1 (7.1)	
Pneumonia	17 (19.1)	16 (37.2)	0.024
Time to onset of pneumonia (day)	3 (2–5)	3 (2–3.5)	0.523
Wound infection	10 (11.2)	3 (7.0)	0.442
Cardiovascular complications	2 (2.3)	0 (0)	0.322
Pleural effusion	5 (5.6)	5 (11.6)	0.221
Chylothorax	4 (4.5)	3 (7.0)	0.551
Recurrent nerve injury	6 (6.7)	3 (7.0)	0.960
Anastomosis stenosis	2 (2.3)	0 (0)	0.322
Myocardial infarction	1 (1.2)	0 (0)	1
Reoperation	2 (2.3)	2 (4.7)	0.450
Death	2 (2.3)	1 (2.3)	0.977

Summary statistics are *n* (%) and median (25th; 75th percentiles)

*IQR* interquartile range

^*^Percentages are calculated by the number of patients with complication (40 in the substernal route group and 15 in the posterior mediastinal route group)

Regarding postoperative pneumonia, all patients were classified as grade II according to Clavien-Dindo classification. Time to onset of postoperative pneumonia was not significantly different between the two groups (3 days vs. 3 days, *p* = 0.523). The results of the multivariate analysis demonstrated that reconstruction route (OR (95% CI), 3.44 (1.41–8.38), *p*-value = 0.007) and operative time (OR (95% CI), 1.01 (1.00–1.01), *p*-value = 0.022) were as independent risk factors for increasing the rate of postoperative pneumonia (Table 4).

**Table 4:** Uni- and multivariable analysis of risk factors for pneumonia

	Univariable analysis			Multivariable analysis		
	No (*N* = 99)	Yes (*N* = 33)	*p*-value	OR	95% CI	*p*-value
Age (years)	59.1 ± 7.4	59.3 ± 8.0	0.915			
Sex			0.400			
Male	94 (75.8)	30 (24.2)				
Female	5 (62.5)	3 (37.5)				
BMI	20.1 ± 3.0	20.5 ± 2.8				
Albumin (g/L)	39.4 ± 3.8	39.5 ± 3.1	0.851			
Hypertension			0.728			
No	75 (75.8)	24 (24.2)				
Yes	24 (72.7)	9 (27.3)				
Diabetes			0.399			
No	88 (74.0)	31 (26.0)				
Yes	11 (84.6)	2 (15.4)				
Cardiovascular disease			0.092			
No	95 (76.6)	29 (23.4)				
Yes	4 (50.0	4 (50.0)				
Chronic kidney disease						
No	99 (75.0)	33 (25.0)				
Yes	0 (0.0)	0 (0.0)				
Chronic lung disease			0.613			
No	90 (75.6)	29 (24.4)				
Yes	9 (69.3.0)	4 (30.8)				
Neoadjuvant therapy			0.206			
No	77 (72.6)	29 (27.4)				
Yes	22 (84.6)	4 (15.4)				
Tumor location			0.683			
Cervical	3 (100.0)	0 (0.0)				
Upper third	4 (80.0)	1 (20.0)				
Middle third	50 (73.5)	18 (26.5)				
Lower third	30 (71.4)	12 (28.6)				
EGJ	12 (85.7)	2 (14.3)				
Histology of disease			0.148			
Squamous cell carcinoma	93 (73.8)	33 (26.2)				
Adenocarcinoma	6 (100.0)	0 (0.0)				
Reconstruction route			0.024	3.44	1.41, 8.38	0.007
Substernal route	72 (80.9)	17 (19.1)				
Post-mediastinal route	27 (62.8)	16 (37.2)				
Pathological T Stage			0.195			
T1	3 (60.0)	2 (40.0)				
T2	22 (91.7)	2 (8.3)				
T3	46 (71.9)	18 (28.1)				
T4	28 (71.2)	11 (28.2)				
Pathological N stage			0.898			
N0	19 (76.0)	6 (24.0)				
N +	80 (74.8)	27 (25.2)				
Operative time	358.6 ± 63.1	381.3 ± 78.2	0.096	1.01	1.00, 1.01	0.022
Abdominal approach			0.842			
Laparoscopy	92 (74.8)	31 (25.2)				
Laparotomy	7 (77.8)	2 (22.2)				

Statistical summary is mean ± standard deviation or *n* (%)

*CI* confidence interval, *OR* odds ratio, *BMI* body mass index

## Discussion

Up till now, the choice of route for gastric conduit reconstruction after MIE was mainly based on the experience and the preference of surgeons, as the available evidence on the optimal route for reconstruction was still controversial. In our study, we analyzed 132 patients with EC who underwent MIE followed by gastric conduit reconstruction with cervical anastomosis. The results demonstrated that the overall postoperative complications were similar between the ST and PM groups. Moreover, there were no significant differences in the rate, date, and severity of anastomotic leakage between the two groups. However, we observed a significantly higher rate of ICU admission and pneumonia in the PM group.

The operative time of the ST group was significantly longer than the PM group. The ST group took additional time to create the route. In fact, the time for route creation of the ST group in our study gradually decreased over time thanks to technical proficiency. In recent cases, the time for making the ST route was about 5–10 min compared to approximately 20 min in the initial cases. The intra-thoracic bleeding resulted from the azygos vein injury during pulling up the gastric conduit through the PM route was a severe complication that only occurred in the PM group. In contrary, this complication could be avoided by using the ST route. Previously, due to its higher morbidity rates including pulmonary complication and anastomotic leakage, the ST route has not been a favorable choice and only considered as an alternative when PM route was not available [15, 25]. However, recent studies demonstrated that the ST route had comparable safety and efficacy to the PM route and thus became a frequent approach for reconstruction after MIE in many centers [8, 11, 14, 16].

Pulmonary complication after MIE, which was a common morbidity with a rate up to 40%, was the main cause of postoperative respiratory failure and mortality [26]. Kunisaki reported the incidence of pneumonia in the ST group was significantly higher than in the PM group [25]; however, this study included open esophagectomy. Meanwhile, the occurrence of this complication was similar between the two routes in most of the other studies [11, 16, 27]. Our study showed the superiority of the ST route in terms of postoperative ICU admission and pneumonia. One possible explanation was that using the ST route could avoid lung compression due to gastric distension.

Cervical anastomotic leakage was one of the most common complications after MIE with a rate of up to 35% [26]. While** s**ome studies had shown that the PM route was associated with a lower occurrence of anastomotic leakage compared to the ST route [15–18], the other revealed no difference [11, 19]. In our study, no gastric conduit necrosis was observed. The rate of anastomotic leakage was similar between the two groups, and most of the cases were classified as minor (84.6% in ST group vs. 64.3% in PM group). Regarding the severity of leakage, grades 2 and 3 seemed to be lower in the ST route but not statistically. Obviously, as the anastomosis in the ST route was placed underneath the cervical incision, the leaking contents could be early drained out when leakage occurred.

Additionally, the reoperation rate was similar between the two groups. Although relatively rare, gastro-tracheobronchial fistula in the thoracic cavity was a fatal complication with a reported rate of 0.3–1.9% [28]. In our study, one patient (2.3%) experienced gastro-bronchial fistula in the PM group. This complication did not occur in the ST group because there was no direct contact between the gastric conduit and the bronchotracheal tract. On top of that, in the ST route, the lower part of the gastric conduit was brought close to the abdominal wall, facilitating the placement of the gastric feeding tube. By avoiding the feeding jejunostomy, we could prevent bowel obstruction at the enterostomy site, as observed in one case of the PM group in our study. Additionally, in the ST group, we routinely closed the esophageal hiatus to avoid a potential hiatus hernia. The benefits of the ST route were also demonstrated in one case of reoperation due to chylothorax, wherein the reoperation to ligate the thoracic duct was relatively simple as there was no presence of the conduit in the surgical field.

This study has several limitations. Firstly, this was a retrospective study, which inherently comes with certain weaknesses. Moreover, the study has an unbalanced sample size in the two groups, which may impact the robustness of the conclusions drawn. Secondly, the long-term outcomes such as anastomotic stricture, gastro-esophageal reflux, quality of life, postoperative radiotherapy, and survival rate were missing.

In conclusion, the ST route was superior to the PM route regarding rates of postoperative ICU admission and pneumonia. Moreover, ST route could avoid some severe postoperative complications that only occurred in the PM route. The ST route can be an alternative for reconstruction after McKeown esophagectomy for EC.

## Data Availability

The data presented in this study are available on request from the corresponding author.

## References

[1] Sung H (2021). Global cancer statistics 2020: GLOBOCAN estimates of incidence and mortality worldwide for 36 cancers in 185 countries. CA Cancer J Clin.

[2] Allemani C (2018). Global surveillance of trends in cancer survival 2000–14 (CONCORD-3): analysis of individual records for 37 513 025 patients diagnosed with one of 18 cancers from 322 population-based registries in 71 countries. Lancet.

[3] Low DE (2019). Benchmarking complications associated with esophagectomy. Ann Surg.

[4] van Workum F (2017). McKeown or Ivor Lewis totally minimally invasive esophagectomy for cancer of the esophagus and gastroesophageal junction: systematic review and meta-analysis. J Thorac Dis.

[5] Biere SS (2012). Minimally invasive versus open oesophagectomy for patients with oesophageal cancer: a multicentre, open-label, randomised controlled trial. Lancet.

[6] Yamasaki M (2015). Impact of the route of reconstruction on post-operative morbidity and malnutrition after esophagectomy: a multicenter cohort study. World J Surg.

[7] van Lanschot JJ (1999). Randomized comparison of prevertebral and retrosternal gastric tube reconstruction after resection of oesophageal carcinoma. Br J Surg.

[8] Yang YS, Niu ZX, Chen LQ (2013). Meta-analysis on reconstructions of posterior mediastinal route and anterior mediastinal route after esophagectomy. Zhonghua Wei Chang Wai Ke Za Zhi.

[9] Wong AC, Law S, Wong J (2003). Influence of the route of reconstruction on morbidity, mortality and local recurrence after esophagectomy for cancer. Dig Surg.

[10] Yasuda T (2012). Ten cases of gastro-tracheobronchial fistula: a serious complication after esophagectomy and reconstruction using posterior mediastinal gastric tube. Dis Esophagus.

[11] Yang J (2016). Esophageal reconstruction: posterior mediastinal or retrosternal route. J Surg Res.

[12] Crespin OM (2016). Hiatal herniation after transhiatal esophagectomy: an underreported complication. J Gastrointest Surg.

[13] Katsoulis IE (2005). Duodenogastric reflux after esophagectomy and gastric pull-up: the effect of the route of reconstruction. World J Surg.

[14] Lv B (2017). Comparison of the outcomes between thoracoscopic and laparoscopic esophagectomy via retrosternal and prevertebral lifting paths by the same surgeon. World J Surg Oncol.

[15] Moremen JR (2017). Substernal reconstruction following esophagectomy: operation of last resort?. J Thorac Dis.

[16] Booka E (2023). What is the best reconstruction procedure after esophagectomy? A meta-analysis comparing posterior mediastinal and retrosternal approaches. Ann Gastroenterol Surg.

[17] Kurahashi Y et al (2021) Anastomosis behind the sternoclavicular joint is associated with increased incidence of anastomotic stenosis in retrosternal reconstruction with a gastric conduit after esophagectomy. Dis Esophagus 34(4):doaa089 10.1093/dote/doaa089 32995867

[18] Chan ML (2011). Reconstruction after esophagectomy for esophageal cancer: retrosternal or posterior mediastinal route?. J Chin Med Assoc.

[19] Horikawa M (2022). Laparoscopic creation of a retrosternal route for gastric conduit reconstruction. Surg Endosc.

[20] Urschel JD (2001). A meta-analysis of randomized controlled trials of route of reconstruction after esophagectomy for cancer. Am J Surg.

[21] Kitagawa Y (2019). Esophageal cancer practice guidelines 2017 edited by the Japan esophageal society: part 2. Esophagus.

[22] Kitagawa Y (2019). Esophageal cancer practice guidelines 2017 edited by the Japan Esophageal Society: part 1. Esophagus.

[23] Clavien PA (2009). The Clavien-Dindo classification of surgical complications: five-year experience. Ann Surg.

[24] Low DE (2015). International consensus on standardization of data collection for complications associated with esophagectomy: Esophagectomy Complications Consensus Group (ECCG). Ann Surg.

[25] Kunisaki C (2007). Appropriate routes of reconstruction following transthoracic esophagectomy. Hepatogastroenterology.

[26] Blencowe N (2012). Reporting of short-term clinical outcomes after esophagectomy a systematic review. Ann Surg.

[27] Zheng YZ (2012). Comparison between different reconstruction routes in esophageal squamous cell carcinoma. World J Gastroenterol.

[28] Mboumi IW, Reddy S, Lidor AO (2019). Complications after esophagectomy. Surg Clin North Am.

